# Predictors and Clinical Impacts of Impaired Heart Rate Variability in Women with Breast Cancer

**DOI:** 10.3390/medicina61040608

**Published:** 2025-03-27

**Authors:** İmran Ceren, Beyza Nur Çitir Durmuşoğlu, Yusuf Ziya Şener, Fadime Bozduman Habip, Sedat Köroğlu, Necla Demir, Öztürk Ateş, Elif Eroğlu Büyüköner

**Affiliations:** 1 Department of Cardiology, Dr Abdurrahman Yurtaslan Ankara Oncology Training and Research Hospital, 06200 Ankara, Türkiye; 2 Department of Internal Medicine, Dr Abdurrahman Yurtaslan Ankara Oncology Training and Research Hospital, 06200 Ankara, Türkiye; 3 Department of Cardiology, Thoraxcenter, Erasmus MC, 3015 GD Rotterdam, The Netherlands; 4 Department of Cardiology, Faculty of Medicine, Kahramanmaraş Sutcu Imam University, 46050 Kahramanmaraş, Türkiye; 5 Department of Medical Oncology, Dr Abdurrahman Yurtaslan Ankara Oncology Training and Research Hospital, 06200 Ankara, Türkiye; 6 Department of Cardiology, Faculty of Medicine, Acibadem University, 34638 İstanbul, Türkiye

**Keywords:** heart rate variability, breast cancer, hypertension, de novo atrial fibrillation

## Abstract

*Background and Objectives*: Breast cancer (BC) is the most prevalent cancer globally, with a significant mortality rate, especially among women. While advances in treatment have reduced BC mortality, cardiovascular complications resulting from anticancer therapies have become a major concern. The autonomic nervous system (ANS) may be affected in BC patients and it is assessed with heart rate variability (HRV). The aim of this study was to investigate the prevalence of impaired HRV, its predictors, and its clinical impacts in BC patients. *Materials and Methods*: We retrospectively screened all female BC patients and enrolled cases who underwent 24 h Holter electrocardiography monitoring with accessible clinical follow-up data. *Results*: This study included 136 BC patients, and the mean age was 56.8 ± 10.8 years old. Impaired HRV was present in 36.8% of patients, and hypertension was identified as a significant predictor of reduced HRV (OR = 3.61, CI: 1.01–12.92, *p* = 0.048). Furthermore, de novo atrial fibrillation (AF) occurred more frequently in patients with impaired HRV (20% vs. 8.1%; *p* = 0.044). None of the HRV parameters were associated with all-cause mortality, and cancer stage was found to be the only independent predictor of all-cause mortality (HR = 3.93, CI: 1.81–8.55; *p* < 0.001). *Conclusions*: HRV is impaired in a significant proportion of patients with BC. Hypertension plays a crucial role in the deterioration of HRV in patients with BC, and de novo AF is more common in patients with impaired HRV. However, HRV does not appear to predict all-cause mortality in patients with BC. This study highlights the importance of the optimal management of cardiovascular risk factors, such as hypertension, to prevent ANS dysfunction in cancer patients.

## 1. Introduction

In 2020, breast cancer (BC) accounted for 2.3 million new cases, representing 11.7% of all new cancer diagnoses. It also resulted in 685,000 deaths, making it the most widespread cancer globally and the leading cause of cancer-related fatalities among women [1,2,3]. While chemotherapy, molecular targeted therapy, and radiotherapy (RT) have significantly reduced BC mortality, cardiovascular damage resulting from anticancer treatments has emerged as a major issue [4,5,6,7].

Alterations in autonomic nervous system (ANS) functions contribute to the etiology and clinical course of cardiovascular disorders induced by BC treatment [8]. Heart rate variability (HRV) is a simple, non-invasive, reliable, and confirmed indicator used to assess the function of the ANS. It has been widely used to evaluate risk in both cardiac and non-cardiac disorders [9,10,11,12,13]. HRV refers to the variation in the time between heartbeats, reflecting the cardiovascular system’s ability to respond to stress. It is an important indicator of cardiac autonomic nervous system function and shows the balance between the sympathetic and parasympathetic systems [14,15,16,17]. 

HRV can be analyzed through two domains: time-domain measures and frequency-domain measures [14,15,16]. High frequency (HF), low frequency (LF), and very low frequency (VLF) represent the frequency domains. In contrast, the time-domain measures include the standard deviation of normal RR intervals (SDNN), the root mean square of successive RR interval differences (RMSSDs), and the percentage of successive RR intervals (pNN50) [9,10]. While HRV indices, such as HF, pNN50, and RMSSD, indicate parasympathetic activity, the LF/HF ratio represents the parasympathetic and sympathetic balance [18]. The SDNN indicates the general autonomic balance [19]. A SDNN > 100 ms is considered normal, a SDNN < 100 ms is considered as moderate impairment, and a SDNN < 50 ms is considered as highly impaired HRV [18]. Numerous studies have shown that HRV is impaired in cancer patients and that impaired HRV is linked to a worse prognosis in BC patients [20,21,22]. Caro-Morán et al. discovered that, one year after treatment, BC patients exhibited lower HRV, cardiovascular imbalances, and vagal nerve damage, in comparison to healthy controls [21]. Giese et al. identified low HF as a risk factor for shorter survival in patients with metastatic or recurrent BC [23].

However, to our knowledge, studies so far have not evaluated the predictors of impaired HRV in BC patients with long-term follow-up. Thus, we aimed to examine and reveal the prevalence of impaired HRV, its predictors, and its impact on clinical outcomes. Additionally, this is the first study to investigate the association between de novo atrial fibrillation (AF) and impaired HRV in BC patients.

Our study differs from others in the literature because the exclusion criteria were broad enough to minimally affect HRV and we also included a larger number of patients and longer-term follow-up compared to previous studies. The mean follow-up period of patients in our study is 61.2 (48.3–82.4) months, and, in this respect, our study is a study that follows patients for a longer period compared to current studies. At the same time, our study includes only symptomatic BC patients and is innovative and different in this respect. Through this study, we hypothesize that by identifying predictors that worsen HRV in BC patients, the risk modification of these factors can be achieved and patient outcomes can be improved.

## 2. Materials and Methods

### 2.1. Study Design and Participants

This study was designed retrospectively and included BC patients who were referred to our cardiology clinic for consultation, with complaints such as palpitations, syncope, and presyncope, between January 2018 and February 2024 and who underwent 24 h Holter electrocardiography (ECG). 

**Figure 1:**
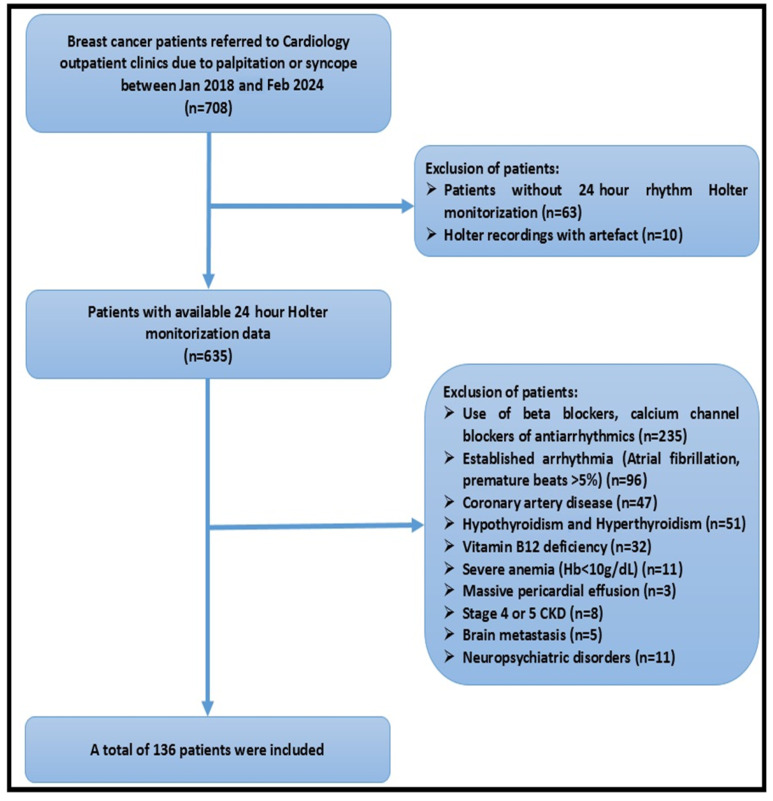
The number and characteristics of the excluded participants.

Patients with brain metastasis, in treatment with drugs related to heart rate (beta blockers, calcium channel blockers, and antiarrhythmic drugs), established AF, ventricular or atrial premature beats > 5%, history of coronary artery disease, massive pericardial effusion, severe anemia (Hb < 10 g/dL), hypothyroidism, hyperthyroidism, vitamin B12 deficiency, Stage 4 or 5 chronic kidney disease, neurologic disorders, and the use of neuropsychiatric drugs were excluded. Finally, 136 BC patients were included. Figure 1 shows the number and characteristics of patients excluded based on the study design. 

### 2.2. Data Collection

Patient data, including sociodemographic characteristics, comorbidities, laboratory results, smoking habits, cancer characteristics, cancer treatments, medications, 12 lead ECG, transthoracic echocardiography findings, clinical follow-up, and mortality information, were obtained from the electronic medical records system.

A multichannel electronic data recorder device and a 24 h ambulatory ECG were used to measure HRV. Devices that would record ambulatory ECG were attached to the patients, and these devices recorded the patients’ ECG for 24 h. The ECG data captured by the ECG recorder (Biomedical Instruments, BI9800TL, Shenzhen, China) were uploaded to a computer with specialized software (Biomedical Instruments, BI, EcgLab, Shenzen, China). Next, the RR interval series was analyzed in both the frequency and time domains over the 24 h period, and they were cleaned using both automated and manual methods to remove ectopic beats and artifacts. The automated filtering was performed using Biomedical Instruments EcgLab software (version 1.0.5.171016). Only data series exhibiting a sinus rhythm in more than 95% of the recorded beats were included in the analysis [24,25,26]. After this filtering and analysis, documented data were obtained from the computer. Time domain HRV analysis included the calculation of SDNN, RMSSD, and pNN50. The frequency domain indices of HRV, including high frequency (HF, 0.15–0.40 Hz), low frequency (LF, 0.04–0.15 Hz), and very low frequency (VLF, 0.003–0.04 Hz), were calculated by spectral analysis of the entire 24 h recording. Spectral analysis was computed using the Fourier transform algorithm [24,26].

The clinical research ethics committee of the Dr. Abdurrahman Yurtaslan Ankara Oncology Training and Research Hospital (Approval Code: 2024-02/15, Approval Date: 22 February 2024) approved this study, which was carried out in compliance with national ethical standards and the Declaration of Helsinki. Due to the nature of the retrospective study, obtaining informed consent from participants was omitted.

### 2.3. Statistical Analyses

SPSS 28.0 statistical package program was used for statistical analyses. Continuous variables were expressed as mean ± standard deviation or median (range) according to their distribution. Categorical variables were presented as percentages. There was almost no missing data, so no data imputation was performed. Logistic regression was used in order to define predictors of decreased SDNN (<100 ms). Numeric variables were changed to categorical variables based on the defined cut-off values obtained from ROC analyses. Each relevant variable was assessed in binary logistic regression and variables with *p* values < 0.200 were proceeded to multinomial logistic regression analysis. Cox regression was performed to identify predictors of all-cause mortality. Variables with *p* values < 0.200 in the univariable Cox regression analysis were further included in the multivariable Cox regression analysis. Multicollinearity assessment was performed for HRV parameters to evaluate correlation with SDNN. Variables with variance influence factor (VIF) higher than 5 were excluded from multivariable Cox model. Kaplan–Meier and log-rank analyses were performed to compare survival between the groups. *p* value less than 0.05 was considered statistically significant.

## 3. Results

### 3.1. Baseline Characteristics

For the study, 6412 female BC patients’ data were screened and 635 patients with a 24 h Holter ECG were examined. A total of 499 of these patients were not included in the study according to the exclusion criteria. Finally, 136 patients with a diagnosis of BC were included in the study. The mean age of the study population was 56.8 ± 10.8 years. The affected breast was the right side in 73 (53.7%) cases, the left side in 56 (41.2%) cases, and bilateral BC was present in 7 (5.1%) cases. A total of 10 (7.4%) patients exhibited evidence of metastatic disease and were classified as stage IV. A total of 31 (22.8%) patients had stage III disease, 67 (49.3%) patients had stage II disease, and 28 (20.6%) patients had stage I disease. A total of 112 (82.4%) patients received anthracycline treatment, 39 (28.7%) patients received HER2-targeted therapy, 84 (61.8%) patients underwent radiotherapy, and 109 (80.1%) patients were treated with hormone therapy. A total of 49 (36%) patients had hypertension, while 16 (11.8%) patients had diabetes. The baseline characteristics of the patients are presented in Table 1.

### 3.2. Prevalence and Predictors of Impaired HRV

The SDNN-based assessment revealed that HRV was highly depressed in three patients (2.2%), moderately depressed in 47 cases (34.6%), and normal in the remaining 86 patients (63.2%).

Binary logistic regression analysis demonstrated that age (OR = 1.03, CI: 1.00–1.07; *p* = 0.029), body mass index (OR = 2.76, CI:1.08–7.05; *p* = 0.001), hypertension (OR = 3.41, CI:1.63–7.14; *p* = 0.001), diabetes (OR = 3.33, CI:1.13–9.82; *p* = 0.029), and the hemoglobin level (OR = 0.71, CI: 0.52–0.99; *p* = 0.043) were predictors of a reduced SDNN (<100 ms) in univariable analyses. The multinomial regression analysis was performed further including the variables with *p*-values < 0.200 in univariable analysis, and only hypertension was associated with a reduced SDNN (OR = 3.61, CI: 1.01–12.92, *p* = 0.048) (Table 2).

### 3.3. De Novo AF and All-Cause Mortality

De novo AF developed in 17 (12.5%) patients. De novo AF occurred significantly more in patients with a reduced SDNN compared to cases with a normal SDNN (20% vs. 8.1%; *p* = 0.044). However, no significant differences were observed in other HRV parameters among cases with and without de novo AF.

**Figure 2:**
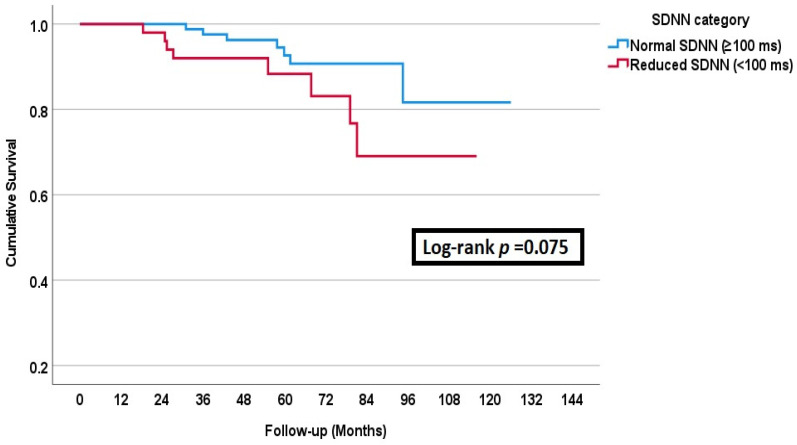
The survival was comparable among the patients with and without impaired HRV.

All-cause mortality occurred in 15 (11%) patients during the follow-up period. Survival analysis revealed no mortality difference between impaired SDNN (<100 ms) and normal SDNN (≥100 ms) groups. Figure 2 illustrates a trend of lower survival in patients with impaired HRV compared to their counterparts with normal HRV (Long-rank *p* = 0.075).

The potential predictors of all-cause mortality were evaluated via univariate Cox regression analysis, and cancer stage, Ca 15-3 level, SDNN index, LF, and VLF were established to be predictors of all-cause mortality. Variables with *p*-values < 0.200 proceeded to the multivariate Cox regression analysis. The multicollinearity assessment was performed via the estimation of the variance influence factor (VIF) for HRV parameters due to the presence of possible correlations across each of them. VIF values of HRV parameters with *p*-values < 0.200 in the univariate analysis were estimated for the SDNN, and parameters with VIF values > 5, (indicating strong correlation, [RMSSD, VIF = 13.8]; [pNN50, VIF = 20.4]; [HF, VIF = 8.4]) were excluded from the multivariate Cox model. Only cancer stage was found to predict all-cause mortality (HR = 3.93, CI: 1.81–8.55; *p* < 0.001) in the multivariate Cox regression model (Table 3).

## 4. Discussion

In this study, we aimed to evaluate the factors causing impaired HRV in BC patients, and we found the following:

(i) Hypertension was the only predictor of reduced SDNN in patients with BC;
(ii) De novo AF occurred significantly more often in BC patients with a reduced SDNN compared to cases with a normal SDNN;
(iii) Although some HRV parameters, including SDNN index, LF, and VLF, were established to be associated with all-cause mortality in univariable analyses, none of the HRV parameters were found to predict all-cause mortality. The cancer stage was established as the only predictor of all-cause mortality.

The most common comorbidity in BC patients is hypertension, and hypertension significantly increases the risk of morbidity and mortality by heightening the cardiotoxic effects of cancer treatments [27]. Braithwaite et al. showed that hypertension is an independent factor that predicts the prognosis in BC [28]. Additionally, studies have shown a strong association between hypertension and the dysfunction of the ANS and that hypertension causes a decrease in HRV [29]. The relationship between autonomic dysfunction and hypertension is bidirectional, as autonomic imbalance can lead to hypertension, while prolonged high blood pressure can worsen autonomic dysfunction [29]. Our study revealed that hypertension is an independent risk factor for HRV deterioration in BC patients. Thus, this study fills an important gap in the literature by demonstrating the relationship between hypertension and HRV in BC patients. These results emphasize the importance of hypertension management in BC patients and demonstrate that the prevention and early diagnosis of hypertension in these patients is critical. Moreover, our results suggest that the effective management of hypertension in BC patients could have significant clinical benefits, particularly in preserving HRV. Monitoring HRV in hypertensive BC patients could serve as an additional marker for assessing cardiovascular risk and tailoring individualized treatment strategies. Given the strong association between hypertension and HRV deterioration, integrating regular HRV monitoring into clinical practice could be an important tool for managing cardiovascular health in BC patients. By identifying HRV abnormalities early, clinicians may be able to adjust treatment regimens to mitigate potential cardiac complications.

AF, the most prevalent arrhythmia, significantly contributes to embolism and cardiovascular deaths, and BC patients with AF exhibit an increased risk of all-cause mortality [30]. Epidemiological studies suggest a bidirectional relationship between BC and AF, with BC patients at higher risk of de novo AF and vice versa [30]. Proposed mechanisms underlying this relationship include inflammation and autonomic dysfunction, with ANS impairment playing a key role [31]. It is well established that impaired cardiac autonomic function, indicated by a significant reduction in HRV, increases the risk of AF onset [32]. Decreased HRV is associated with sympathetic nervous system overstimulation and decreased vagal tone, which may lead to cardiac arrhythmias and increased inflammation, contributing to both the development of AF and the progression of BC [33]. An imbalance between sympathetic and parasympathetic activity has been linked to AF, and individuals with cancer frequently show signs of autonomic dysfunction [34]. Furthermore, treatments for BC, including surgery, chemotherapy, and radiotherapy, can contribute to the development of AF [30]. Chemotherapy drugs commonly used for BC, such as alkylating agents, anthracyclines, paclitaxel, and trastuzumab, are cardiotoxic and can trigger AF [30]. Additionally, pain medications, like non-steroidal anti-inflammatory drugs and opioids, often used by advanced cancer patients are linked to a higher risk of AF [30]. Moreover, pain and stress in cancer may also increase sympathetic nervous activity, making individuals more prone to AF [34]. Inflammation, like autonomic dysfunction, is one of the pathophysiological mechanisms that help explain the link between cancer and AF. [34]. Clinical studies have demonstrated elevated levels of pro-inflammatory markers in both AF and cancer, and several risk factors contribute to both conditions [34]. In summary, BC patients have autonomic dysfunction and reduced HRV, and reduced HRV is associated with an increased risk of developing AF [33]. Therefore, treatment approaches that improve HRV may play an important role in the management of both AF and BC, improving patient outcomes by reducing cardiovascular risks.

In another context, hypertension is a key risk factor for AF and is associated with significantly reduced HRV compared to normotensive individuals [35,36]. Our findings indicate a higher incidence of de novo AF in BC patients with impaired HRV, highlighting hypertension’s dual role in AF risk—both directly and via HRV reduction. These findings underscore the importance of hypertension management in mitigating de novo AF in BC patients.

In general, the results regarding HRV parameters reveal lower parasympathetic regulation, which is associated with poorer survival [20]. A recent study has shown that SDNN, VLF, and LF are associated with tumor stage, and HRV indices could predict tumor staging in BC patients [37]. In another study, Giese et al. found that low HF was a risk factor for shorter survival in patients with metastatic or recurrent BC [23]. In our study, while in the univariate analysis, the SDNN index, LF and VLF predicted all-cause mortality; in the multivariate Cox regression model, only the cancer stage was found to predict all-cause mortality. There are several explanations for these controversial results. First, in contrast to other studies, our study included only symptomatic patients and used strict exclusion criteria, which resulted in the exclusion of patients with a higher comorbidity burden. The majority of patients enrolled had early-stage disease, which may have resulted in a lower rate of impaired HRV due to less exposure to chemotherapy and radiotherapy, which may have further led to lower mortality rates. Finally, we could not assess the effect of HRV on cardiovascular mortality due to lack of data on cause of death. Changes in HRV may be more related to cardiovascular death.

Reduced HRV is an indicator of autonomic dysfunction, and autonomic dysfunction has been associated with many cardiovascular diseases, such as heart failure, hypertension, arrhythmias, and myocardial ischaemia [38]. Studies have shown that BC patients with reduced HRV are at increased risk of these cardiovascular manifestations, suggesting vagal dysfunction [33]. Impaired HRV emphasizes the importance of considering the increased cardiovascular risk in the long-term follow-up of these patients. In particular, the early diagnosis and management of long-term cardiovascular complications that may develop in patients with lower HRV may improve patient outcomes.

### Limitations

Our study cohort does not include any patients with poor performance status who are generally not candidates for chemotherapy regimens and are mostly followed in palliative care units, as we only screened patients admitted to outpatient cardiology clinics and we might have missed these patients. The adverse effects of RT may appear late, even 10 years after RT. Therefore, our follow-up period may be too short to demonstrate the late effects of RT. Pain is a challenging problem in patients with cancer and pain contributes to changes in the ANS. We did not assess patients’ pain status which also might have an impact on HRV parameters. In our study, the exclusion criteria were kept very broad in order to minimize the effect on HRV, and those using beta blockers or other antiarrhythmic drugs that could affect HRV were not included in the study. This situation constitutes the strength of our study. However, this feature limits the generalizability of the findings to various patient populations and treatment protocols. This retrospective study was conducted without a healthy volunteer control group. In fact, since many studies have previously shown that HRV is reduced in BC patients compared to healthy controls, it was thought that planning a control group as a design would not provide additional significant information. Rather, our aim in this study was to identify predictors of impaired HRV in BC patients and to reveal its long-term clinical outcomes. Other limitations of our study are its retrospective and single-center design. Considering the limitations encountered during this study, prospective and multicenter studies can be conducted in the future to validate the results.

## 5. Conclusions

This study showed that hypertension is an independent risk factor for impaired HRV in BC patients and that de novo AF develops more frequently in BC patients with impaired HRV. The optimal management of cardiovascular risk factors, such as hypertension, will reduce the risk of ANS dysfunction in BC patients and improve patient outcomes. 

## Figures and Tables

**Table 1:** Baseline characteristics of the study population.

	All Patients (*n* = 136)	Normal SDNN (*n* = 86)	Reduced SDNN (*n* = 50)	*p*-Value	Missing (%)
**Age, years**	56.8 ± 10.8	55.2 ± 10.8	59.5 ± 10.3	0.013 *	0
**Body mass index, kg/m^2^**	28.7 ± 6.8	27.5 ± 5.6	30.6 ± 8.1	0.044 *	39
**Smoking history, *n* (%)**	50 (36.8)	34 (39.5)	16 (32)	0.380	0
**Indication for Holter monitorization**				0.332	0
- Palpitation	113 (83.1)	74 (86)	39 (78)		
- Presyncope and syncope	23 (16.9)	12 (14)	11 (22)		
**Comorbidities, *n* (%)**					
Hypertension	49 (36)	22 (25.6)	27 (54)	<0.001 *	0
Diabetes	16 (11.8)	6 (7)	10 (20)	0.046 *	0
**Cancer characteristics**					
Stage				0.853	0
- Stage I	28 (20.6)	16 (18.6)	12 (24)		
- Stage II	67 (49.3)	43 (50)	24 (48)		
- Stage III	31 (22.8)	21 (24.4)	10 (20)		
- Stage IV	10 (7.4)	6 (7)	4 (8)		
Grade				0.706	0.07
- Grade I	18 (13.3)	11 (12.8)	7 (14.3)		
- Grade II	60 (44.4)	40 (46.5)	20 (40.8)		
- Grade III	56 (41.5)	34 (39.5)	22 (44.9)		
- Grade IV	1 (0.7)	1 (1.2)	0 (0)		
Side of the cancer				0.181	0
- Right	73 (53.7)	41 (47.7)	32 (64)		
- Left	56 (41.2)	40 (46.5)	16 (32)		
- Bilateral	7 (5.1)	5 (5.8)	2 (4)		
ECOG category				0.263	0
- ECOG 0	69 (50.7)	40 (46.5)	29 (58)		
- ECOG I	61 (44.9)	43 (50)	18 (36)		
- ECOG II	6 (4.4)	3 (3.5)	3 (6)		
- ECOG III–IV	0 (0%)	0 (0)	0 (0)		
Cancer treatment, *n* (%)					
- Anthracycline therapy	112 (82.4)	70 (81.4)	42 (84)	0.880	0
- HER2-targeted therapy	39 (28.7)	25 (29.1)	14 (28)	0.894	0
- Radiotherapy (thoracal)	84 (61.8)	57 (66.3)	27 (54)	0.200	0
- Hormonotherapy	109 (80.1)	71 (82.6)	38 (76)	0.483	0
Anthracycline cardiotoxicity, *n* (%)	4 (2.9)	3 (3.5)	1 (2)	1.000	0
**Medications (Cardiovascular), *n* (%)**					
RAS inhibitors	47 (34.6)	20 (23.3)	27 (54)	<0.001	0
Statins	8 (5.9)	1 (1.2)	7 (14)	0.004 *	0
Antiplatelet treatment	17 (12.5)	5 (5.8)	12 (24)	0.003 *	0
**Echocardiographic measurements**					
LA diameter, mm	35.8 ± 3.2	35.1 ± 2.9	36.9 ± 3.5	0.002 *	0
LVEDD, mm	44.0 ± 1.9	43.6 ± 2.0	44.6 ± 1.7	0.004 *	0
LVEF, %	60.4 ± 3.0	60.3 ± 3.6	60.5 ± 1.9	0.749	0
**Laboratory parameters**					
Hemoglobin, g/dL	12.8 ±1.1	13.0 ± 0.9	12.6 ± 1.3	0.040 *	0
Creatinine, mg/dL	0.68 ± 0.16	0.66 ± 0.14	0.71 ± 0.18	0.099	0
TSH, IU/L	1.85 (1.23–2.78)	1.73 (1.14–2.83)	2.11 (1.44–2.80)	0.041 *	0
Vitamin B12, pg/mL	319 (235–453)	301 (229–456)	330 (2448–455)	0.075	0
Ca 15-3	15.3 (10.4–22.0)	15.2 (9.4–22.6)	16.0 (12.3–20.9)	0.896	0
**Heart rate variability parameters**					
Mean heart rate, bpm	77.6 ± 10.2	74.6 ± 8.4	82.7 ± 11.1	<0.001 *	0
SDNN, ms	114 (87–144)	137 (116–160)	81 (72–90)	<0.001 *	0
SDNN index	46 (37–56)	50 (44–66)	36 (29–44)	<0.001 *	0
pNN50	6 (2–11)	8 (4–16)	2.5 (1–5)	<0.001 *	0
RMSSD, ms	27 (23–35)	30 (26–40)	23 (21–28)	<0.001 *	0
Triangular index	23 (18–29)	27 (21–33)	18 (14–22)	<0.001 *	0
HF, ms^2^	138 (85–226)	161 (118–351)	94 (45–151)	<0.001 *	0
LF, ms^2^	280 (150–448)	378 (266–563)	145 (94–240)	<0.001 *	0
VLF, ms^2^	631 (392–786)	699 (534–824)	406 (300–552)	<0.001 *	0
LF/HF	1.96 (1.26–2.71)	2.02 (1.33–3.00)	1.78 (1.13–2.33)	<0.001 *	0
**Follow-up, months**	61.2 (48.3–82.4)	65.8 (53.3–83.3)	54.9 (40.3–79.0)	0.075	0

Abbreviations: bpm, beat per minute; ECOG, Eastern Cooperative Oncology Group; HER2, Human epidermal growth factor receptor-2; RAS, renin angiotensin system; LA, left atrium; LVEF, left ventricular ejection fraction; LVEDD, left ventricular end-diastolic diameter; TSH, thyroid stimulating hormone; SDNN, standard deviation of NN intervals; RMSSD, root mean square of successive differences; pNN50: percentage of successive normal interbeat intervals greater than 50 ms; HF, high frequency; LF, low frequency; and VLF, very low frequency. * *p*-values < 0.05 indicate statistical significance.

**Table 2:** Univariable and multivariable logistic regression analysis for reduced SDNN (<100 ms).

Variable	OR (CI)	*p*-Value	OR (CI)	*p*-Value
Age, (>58 years)	1.03 (1.00–1.07)	0.029 *	0.71 (0.19–2.57)	0.603
BMI, (>27)	2.76 (1.08–7.05)	0.033 *	1.88 (0.67–5.26)	0.963
left chest radiotherapy	0.58 (0.31–1.085)	0.089	0.83 (0.25–2.7131)	0.833
Stage	0.89 (0.58–1.36)	0.602		
ECOG score	0.76 (0.41–1.40)	0.386		
Anthracycline therapy	1.20 (0.47–3.04)	0.701		
HER2-targeted therapy	0.94 (0.43–2.05)	0.894		
Hormonotherapy	0.66 (0.28–1.57)	0.357		
Hypertension	3.41 (1.63–7.14)	0.001 *	3.61 (1.01–12.92)	0.048 *
Diabetes mellitus	3.33 (1.13–9.82)	0.029 *	1.04 (0.16–6.49)	0.963
Hemoglobin, (<12.6)	0.71 (0.52–0.99)	0.043 *	0.75 (0.26–2.15)	0.604
Creatinine, (>0.68)	5.84 (0.69–49.15)	0.104	1.22 (0.43–3.43)	0.702
Vitamin B12	1.00 (0.99–1.00)	0.363		

Abbreviations: BMI, Body mass index; ECOG, Eastern Cooperative Oncology Group; and HER2, Human epidermal growth factor receptor-2. * *p*-values < 0.05 indicate statistical significance.

**Table 3:** Univariable and multivariable Cox regression analysis for all-cause mortality.

Variable	HR (CI)	*p*-Value	HR (CI)	*p*-Value
Left-sided cancer	0.41 (0.13–1.30)	0.132	0.67 (0.18–2.46)	0.550
Stage	4.05 (2.14–7.66)	<0.001 *	3.93 (1.81–8.55)	<0.001 *
ECOG score	1.90 (0.82–4.43)	0.133	1.15 (0.40–3.25)	0.791
Ca 15-3	1.00 (1.002–1.003)	<0.001 *	1.00 (0.99–1.00)	0.901
Hypertension	2.75 (0.98–7.74)	0.055	3.54 (0.99–1.00)	0.051
SDNN	0.98 (0.97–1.00)	0.053	1.00 (0.98–1.03)	0.732
SDNN index	0.95 (0.91–0.98)	0.009 *	0.96 (0.90–1.03)	0.228
RMSSD ^#^	0.92 (0.84–1.00)	0.051		
pNN50 ^#^	0.93 (0.84–1.02)	0.159		
HF, ms^2 #^	0.99 (0.99–1.00)	0.120		
LF, ms^2^	0.99 (0.99–1.00)	0.041 *	1.00 (0.99–1.00)	0.978
LF/HF	0.86 (0.50–1.47)	0.588		
VLF, ms^2^	0.99 (0.99–1.00)	0.022 *	0.96 (0.90–1.03)	0.835

Abbreviations: ECOG, Eastern Cooperative Oncology; SDNN, standard deviation of NN intervals; RMSSD, root mean square of successive differences; pNN50: percentage of successive normal interbeat intervals greater than 50 ms; HF, high frequency; LF, low frequency; and VLF, very low frequency. ^#^ Variables were excluded from multivariable Cox model due to having high VIF values for correlation with SDNN, despite their *p*-values < 0.200 in univariable Cox regression. * *p*-values < 0.05 indicate statistical significance.

## Data Availability

The datasets utilized in this investigation are not publicly available due to institutional rules and patient confidentiality concerns.
